# Factors associated with arthrofibrosis-related revision following 14,325 total or unicompartmental knee arthro-plasties: an analysis from the Dutch Arthroplasty Registry

**DOI:** 10.2340/17453674.2024.41988

**Published:** 2024-10-15

**Authors:** Myrthe P F VAN DE VEN, Joris BONGERS, Anneke SPEKENBRINK-SPOOREN, Sander KOËTER

**Affiliations:** 1 Department of Orthopaedic Surgery, Canisius Wilhelmina Ziekenhuis, Nijmegen; 2 Department of Orthopaedic Surgery, Radboud Institute for Health Sciences, Radboud University Medical Centre, Nijmegen; 3 Dutch Arthroplasty Registry (Landelijke Registratie Orthopedische Interventies), ‘s-Hertogenbosch, The Netherlands

## Abstract

**Background and purpose:**

Arthrofibrosis is a fibrotic joint disorder that can impair the results of knee arthroplasty surgery by limiting the range of motion, functionality, and quality of life. We aimed to investigate whether patient and procedural characteristics are associated with arthrofibrosis-related revision following unicompartmental and total knee arthroplasty (UKA and TKA).

**Methods:**

A prospective observational study was conducted using data from the Dutch Arthroplasty Registry. We included 14,325 revisions performed in 2014–2022 following primary knee arthroplasty. Demographic and surgical characteristics including age, sex, BMI, smoking status, and prosthesis type (TKA versus UKA) were analyzed. Multiple logistic regression was performed to investigate associations between these factors and arthrofibrosis-related revisions, compared with other reasons.

**Results:**

Revisions were due to arthrofibrosis in 711 (5%) patients. There were significantly higher associations for younger age (odds ratio [OR] 0.97, 95% confidence interval [CI] 0.96–0.97)), male sex (OR 1.2, CI 1.0–1.4), lower BMI (OR 0.97, CI 0.95–0.98), non-smoking status (OR 1.7, CI 1.2–2.3), and TKA (OR 7.7, CI 5.2–12), for arthrofibrosis-related revision compared with any other reason for revision.

**Conclusion:**

Younger patients, men, non-smokers, patients with a lower BMI, and those who had primary TKA were more often associated with revision due to arthrofibrosis than other reasons for revision.

Arthrofibrosis is a joint disorder that is defined by excessive collagen production and adhesions, resulting in limited joint motion and pain [1]. It is a known complication following total knee arthroplasty (TKA) with a prevalence ranging between 1.3% and 60% [2-4]. As arthrofibrosis accounts for 28% of surgical hospital readmission within 90 days following TKA, it causes a substantial economic and societal burden [5-7]. It also has a significant impact on patient satisfaction, with inability to perform important daily tasks after TKA [8].

Developing arthrofibrosis after TKA has been attributed to a multifactorial nature, including a dysregulated inflammatory response [9,10]. This can be separated into patient-specific and procedure-specific factors. Patient-specific factors include age, biological sex, body mass index (BMI), patient comorbidities, intoxications, and poor baseline range of motion (ROM) of the affected joint prior to the knee arthroplasty. Procedure-related factors encompass the type of implant, technical factors such as the sizing of components, malrotation of components, and failure to balance sagittal gaps [9].

Little is known about the patient and procedural factors in the population requiring revision TKA due to arthrofibrosis. It might be that specific patient groups with specific procedural characteristics are more likely to undergo revision for this reason than other patients. A deeper understanding of this matter could support clinical decision-making. Therefore, we aimed to investigate whether patient and procedural characteristics are associated with arthrofibrosis-related revision.

## Methods

### Dutch Arthroplasty Register (LROI)

This is an observational study, using data from the Dutch Arthroplasty Registry (LROI: Landelijke Registratie Orthopedische Interventies). The LROI has had a completion rate of 93–100% for primary and revision knee arthroplasty since 2014 [11]. Smoking status, BMI, and arthrofibrosis have been registered since 2014. Smoking status is categorized as being smoker or non-smoker, based on self-reported smoking behavior. No information is available regarding smoking history. Arthrofibrosis is defined by the LROI as an inflammatory condition leading to excessive connective tissue production, resulting in limited ROM of the knee [12]. Revisions are defined as any change (insertion, replacement, and/or removal) of 1 or more components of the prosthesis. Reasons for revision are registered by the surgeon, and more than 1 reason for revision can be registered. Diagnoses were not validated.

This study is reported according to STROBE guidelines.

### Data selection

All first revision procedures after a TKA or UKA between 2014 and 2022 in the Netherlands were included. Type of primary prosthesis and patient characteristics including age, biological sex, BMI, American Society of Anesthesiologists (ASA) score, smoking status, and reasons for revision as registered at the revision procedure were selected from the LROI database. Ages < 10 and > 110 years and BMI < 10 and > 70 were reported as missing values.

### Statistics

Demographic and surgical characteristics in patients who underwent knee revision surgery were assessed using descriptive analyses. Values are presented as n (%) and mean (standard deviation [SD]).

**Figure 1:**
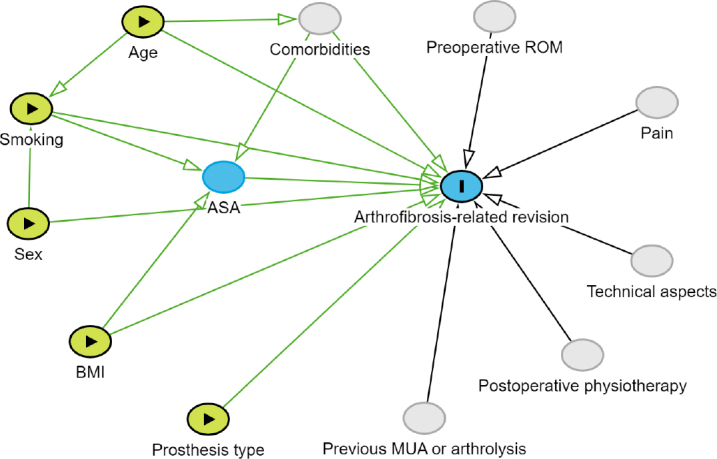
Directed acyclic graph (DAG) with arthrofibrosis-related revision as outcome (marked in blue with **I**). Exposure variables are marked in yellow with a triangle (►). The mediator variable is depicted in blue. Unmeasured variables are depicted in gray.

In a multiple logistic regression model, we tested whether differences in biological sex, age, BMI, smoking status, and type of prosthesis were associated with revisions for arthrofibrosis, compared with revisions for any other reason. In the model, males (versus females), age (years), BMI, non-smoking (versus smoking), and TKA (versus UKA) were reference categories. Regression coefficients are presented as odds ratios (ORs) with 95% confidence interval (CI). A directed acyclic graph (DAG) was created to identify possible confounders that should be accounted for (Figure 1). The confounders age and sex were adjusted for in the model. ASA score was not included, as this was identified as mediator.

Statistical analysis was done using R version 4.1.3. (RStudio Team [2020], RStudio: Integrated Development for R, Boston, USA) [13]. Statistical significance was set at 0.05.

### Ethics, funding, data sharing, and disclosures

Ethical approval for the current study was not applicable according to the Dutch Medical Research Involving Human Subjects Act. The study was approved by the institutional Scientific Advisory Board of the LROI (LROI2023-116). Data was made available by the LROI. However, restrictions apply, and data was under license for the current study. The study protocol can be provided by the authors upon request. No funding was received for this study. There were no conflicts of interest. Complete disclosure of interest forms according to ICMJE are available on the article page, doi: 10.2340/17453674.2024.41988

## Results

### Baseline demographics

**Figure 2:**
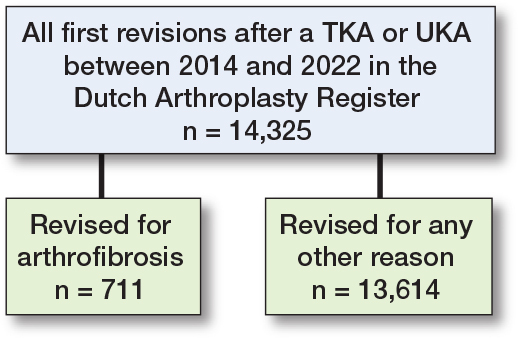
Patient flow chart.

14,325 revisions in 14,089 patients were included in the analysis (Figure 2). Demographic and surgical characteristics showed that, overall, patients had a mean age of 67 (9.4) years, a BMI of 30 (5.1), were mostly female (62%), had ASA class II (63%), were non-smokers (88%), and had primary TKA (81%). Incidence of arthrofibrosis as reason for revision was 5% (n = 711). The most common reason for revision in general was instability (27%), followed by patellar pain (26%). Values separated by arthrofibrosis as reason for revision versus other reasons for revision are displayed in Table 1.

**Table 1:** Demographic and surgical characteristics. Values are count (%) unless otherwise specified

Factor	Arthrofibrosis as reason for revision	
	Yes	No
	(n = 711)	(n = 13,614)
Sex **^a^**		
Male	304 (43)	5,146 (38)
Female	406 (57)	8,454 (62)
Missing	1	14
Age, mean (SD) **^a^**	65 (9.0)	67 (9.4)
BMI, mean (SD) **^b^**	29 (5.0)	30 (5.1)
ASA class		
I	77 (11)	1,365 (10)
II	500 (70)	8,458 (62)
III–IV	121 (17)	3,604 (27)
Missing	13	187
Smoking		
No	650 (91)	11,986 (88)
Yes	48 (6.8)	1,261 (9.3)
Missing	13	367
Type of prosthesis		
TKA	685 (96)	10,926 (80)
UKA	26 (3.7)	2,688 (20)
Reason for revision **^c^**		
Arthrofibrosis	711 (100)	–
Infection	41 (5.8)	2,425 (18)
Patellar dislocation	14 (2.0)	339 (2.5)
Patellar pain	180 (25)	3,505 (26)
Wear inlay	12 (1.7)	568 (4.2)
Periprosthetic fracture	6 (0.8)	348 (2.6)
Malalignment	127 (18)	1,682 (12)
Instability	82 (12)	3,735 (27)
Loose femur	41 (5.8)	866 (6.4)
Loose tibia	98 (14)	2,744 (20)
Loose patella	6 (0.8)	135 (1.0)
Progressive osteoarthritis	9 (1.3)	1,026 (7.5)
Bearing dislocation **^d^**	3 (0.4)	68 (0.5)
Other	65 (9.1)	1,276 (9.4)

BMI = body mass index; ASA = American Society of Anesthesiologists; TKA = total knee arthroplasty; UKA = unicompartmental knee arthroplasty.

a < 0.2% missing for age.

b < 3% missing for BMI.

c 1 patient may have more than 1 reason for revision. As such, the total percentage is over 100%.

d Please note: bearing dislocation was not registered before 2022.

### Associations with arthrofibrosis as reason for revision

Male sex, younger age, lower BMI, non-smoking, and primary TKA had significantly higher associations for arthrofibrosis-related revision compared with revision for any other reason (Table 2).

**Table 2:** Associations of demographic and surgical variables between arthrofibrosis-related revision and other reasons for revision

Factor	Adjusted OR (CI)
Male sex	1.2 (1.0–1.4)
Age	0.97 (0.96–0.97)
BMI	0.97 (0.95–0.98)
Non-smoking	1.7 (1.3–2.3)
TKA	7.8 (5.3–12)

OR = odds ratio; CI = 95% confidence interval; see Table 1 for other abbreviations.

## Discussion

We aimed to investigate whether patient and procedural characteristics were associated with arthrofibrosis-related revision and found that patients undergoing revision for arthrofibrosis generally were of younger age, male sex, had a lower BMI, non-smoking status, and primary TKA compared with patients with other reasons for revision.

A recent study also identified younger age as a factor [14]. They reported an OR of 2.0 (CI 1.4–2.7) for patients < 65 years versus ≥ 65 years. They found no significant results for the association with sex for arthrofibrosis-related revision. This could be due to heterogeneity of the data from the several registries they used. Other known risk factors for revision arthroplasty in general, such as BMI and smoking, have not yet been associated with arthrofibrosis-related revision arthroplasty in the current literature [15]. Nonetheless, a recent study reported similar incidences of arthrofibrosis-related revision in smokers and non-smokers, but the association between smoking and arthrofibrosis was not assessed [16].

It remains unclear what the pathophysiology is for the development of arthrofibrosis after knee arthroplasty [17]. It is known, however, that there is a lack of apoptosis, which influences the normal healing process, leads to pathologic scar formation, and creates a fibrotic state [17,18]. The differences between UKA and TKA can possibly be explained by the differences in scar formation, due to the differences in surgical approach to the knee (i.e., the size of the surgical trauma and the necessity for patella mobilization or eversion). The associations between smoking, sex, BMI, and arthrofibrosis remain unexplained. Possibly, maximum flexion is limited through earlier thigh–calf contact in patients with a higher BMI. This might explain why arthrofibrosis as reason for revision was less prevalent in patients with a higher BMI.

There are several treatment options available to treat arthrofibrosis following knee arthroplasty, depending on its etiology and chronicity [19]. Initially, treatment is started with intensive physiotherapy with or without continuous passive motion devices [17]. If this does not suffice to increase ROM, manipulation under anesthesia (MUA) or arthrolysis (open or arthroscopic) are considered [17]. If all of these interventions fail, revision arthroplasty may be warranted [9,10,17].

Intensive postoperative physiotherapy has been found to be the most important factor for good flexion ability [20]. To prevent arthrofibrosis, (i) management of inflammation, pain, and swelling, (ii) frequent monitoring, and (iii) maintaining or restoring ROM deficits should be prioritized over building quadriceps muscle strength in the first postoperative stage [17]. Studies have found that being male or of younger age increased the odds of not showing up at initial physiotherapy appointments [21,22]. This could increase the possibility of developing arthrofibrosis. Therefore, patients with these demographics, as well as with a low BMI, non-smokers, and TKA, should be more closely monitored and educated after initial knee arthroplasty for prevention of developing arthrofibrosis.

### Strengths

We collected data prospectively, with a very high completeness of registration [11,23]. Furthermore, this was data from a national registry, which provides generalizable results for Dutch TKA patients. Additionally, we could investigate a large cohort of patients who underwent revision arthroplasty due to arthrofibrosis (n = 711), whereas a comparable study by Rockov et al. included only 42 cases [6].

### Limitations

The main limitation of this study was the cross-sectional design, which renders causative interpretations impossible. Furthermore, we only had access to data from patients who underwent revision arthroplasty and had not benefited from MUA or arthrolysis. Missing variables that were not available in the LROI registry could potentially be associated with arthrofibrosis-related revision [24], as shown in the DAG. For example, we did not have data regarding diagnosis or previous knee surgeries (such as MUA, open or arthroscopic lysis) before the revision arthroplasty. Other relevant factors include comorbidities, pain medication, perioperative technical aspects (surgery time, implant brand, sizing and malrotation of components, failure to balance sagittal gaps, cruciate retaining vs posterior stabilized, mobile bearing vs fixed bearing), and postoperative physiotherapy. There was also no data available on pre- and postoperative ROM. Last, the reasons for revision were not validated and relied on the surgeon’s report. However, the incidence of arthrofibrosis as reason for revision in this cohort was 5.0%, comparable to Lewis et al. (4.1%) and Schroer et al. (7.0%) [14,25]. The demographics of the patients requiring revision due to arthrofibrosis were comparable to the study by Rockov et al., where the authors included only patients requiring revision TKA due to arthrofibrosis [6]. This suggests that the classification of arthrofibrosis as reason for revision in our study was likely valid.

### Conclusion

Younger patients, men, non-smokers, patients with a lower BMI, and those who had primary TKA were more often associated with revision due to arthrofibrosis than other reasons for revision.

In perspective, these factors could be considered during clinical decision-making and expectation management of patients with osteoarthritis.

## Notes

MV and SK conceived and designed the study. AS collected the database. MV performed the analyses. MV, JB, AS, and SK helped with the interpretation of the results. MV wrote the manuscript, while JB, AS, and SK adjusted the manuscript.

Handling co-editors: Marianne Westberg and Philippe Wagner

*Acta* thanks Mona Badawy and Anders Troelsen for help with peer review of this manuscript.
